# GBT1118, a Voxelotor Analog, Ameliorates Hepatopathy in Sickle Cell Disease

**DOI:** 10.3390/medicina60101581

**Published:** 2024-09-26

**Authors:** Elio Haroun, Seah H. Lim, Dibyendu Dutta

**Affiliations:** Division of Hematology and Oncology, Department of Medicine, State University of New York Upstate Medical University, Syracuse, NY 13210, USA; haroune@upstate.edu (E.H.); limse@upstate.edu (S.H.L.)

**Keywords:** GBT1118, sickle cell disease, iron overload, hepatopathy, ferroptosis, liver injury

## Abstract

*Background and Objectives:* In sickle cell disease (SCD), hepatopathy is a cumulative consequence of ischemia/reperfusion (I/R) injury from a vaso-occlusive crisis, tissue inflammation, and iron overload due to blood transfusion. Hepatopathy is a major contributing factor of shortened life span in SCD patients. We hypothesized that the voxelotor, a hemoglobin allosteric modifier, ameliorates sickle hepatopathy. *Materials and Methods:* Townes SCD mice and their controls were treated with either chow containing GBT1118, a voxelotor analog, or normal chow. We evaluated inflammation, fibrosis, apoptosis and ferroptosis in their livers using qPCR, ELISA, histology, and immunohistochemistry. *Results:* GBT1118 treatment resulted in reduced hemolysis, iron overload and inflammation in the liver of SCD mice. There were significant reductions in the liver enzyme levels and bile acids. Furthermore, GBT1118-treated mice exhibited reduced apoptosis, necrosis, and fibrosis. Increased ferroptosis as evident from elevated 4-hydroxynonenal (4-HNE) staining, *malondialdehyde* (MDA) levels, and expression of *Ptgs2* and *Slc7a11* mRNAs, were also significantly reduced after GBT1118 treatment. To explain the increased ferroptosis, we evaluated iron homeostasis markers in livers. SCD mice showed decreased expression of heme oxygenase-1, ferritin, hepcidin, and ferroportin mRNA levels. GBT1118 treatment significantly increased expressions of these genes. *Conclusions:* Our results suggest GBT1118 treatment in SCD confers the amelioration of sickle hepatopathy by reducing inflammation, fibrosis, apoptosis, iron overload and ferroptosis.

## 1. Introduction

Sickle cell disease (SCD) is a monogenetic disorder in which a point mutation occurs in the sixth codon of the β-globin gene for hemoglobin (Hb), resulting in a substitution of the hydrophilic glutamic acid residue (Glu) by a hydrophobic valine residue (Val). The mutant Hb, sickle hemoglobin (HbS), polymerizes upon deoxygenation, rendering rigidity to the erythrocytes and causing the erythrocytes to assume a sickle morphology. As a result, SCD patients suffer intermittent microvascular occlusion causing vaso-occlusive crisis (VOC) and premature hemolysis. Repeated VOC-induced local tissue ischemia and production of excess heme due to chronic intravascular hemolysis contribute to inflammation, iron overload, and progressive organ damage [1]. This predisposes SCD patients to multiple chronic conditions [2], functional limitations, poor quality of life, and shortened life span [3]. Hepatopathy is one of the chronic conditions that contribute to early mortality in SCD patients [4]. Approximately 30% of autopsies in SCD patients showed liver cirrhosis indicative of chronic liver disease [5]. While blood transfusion aims to reduce sickling-related complications such as acute chest syndrome (ACS) and stroke [6], it has limited benefits in ameliorating sickle hepatopathy [7].

Voxelotor, an allosteric Hb modifier, covalently and reversibly binds to the N-terminal Val of the Hb alpha chain to improve Hb-O_2_ affinity and arterial oxygen loading to inhibit HbS polymerization [8]. In the Hemoglobin Oxygen Affinity Modulation to Inhibit HbS Polymerization (HOPE) trial, SCD patients in the voxelotor arms had a reduction in hemolysis, with the consequence of increases in Hb concentrations, compared to the placebo group [9]. The voxelotor has been approved by both the United States Food and Drug Administration (FDA) and the European Medicines Agency (EMA) to treat SCD. GBT1118, an analog of voxelotor, was designed to investigate the effect of the voxelotor in transgenic SCD mice [10]. It was incorporated into the laboratory chow to enable administration of the drug to mice without oral gavages. Consumption of GBT1118-containing chow results in a 30% Hb occupancy in mice, comparable to voxelotor-treated patients [10]. GBT1118 also has better pharmacokinetic properties [10]. It improves oxygen delivery to hypoxic tissues in mouse models of hypoxic tissue injury [11,12], protects from renal damage [13] and improves intestinal pathophysiology in SCD mice [14]. In this current study, we investigated whether GBT1118 use is associated with protection against sickle hepatopathy. Here, we show that the reduction in the availability of excess heme due to reduced hemolysis in SCD after GBT1118 treatment restored iron homeostasis in the liver. This was associated with an improvement in liver function and a decrease in hepatic iron overload, ferroptosis, inflammation, apoptosis, and fibrosis.

## 2. Materials and Methods

### 2.1. Mice

**Figure 1:**
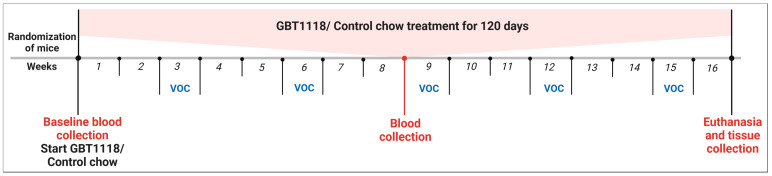
Treatment schema. Non-SCD or SCD Townes were randomized and treated either with control chow or chow containing GBT1118 for 120 days. Mice were kept under strict water deprivation for 12 h for seven consecutive nights every three weeks starting from Week 3 to induce VOC.

Six-week-old male Townes SCD mice (homozygous for *Hba^tm1(HBA)Tow^* and homozygous for Hbb^tm2(HBG1,HBB*)Tow^) and their aged-matched non-sickling controls (homozygous for *Hba^tm1(HBA)Tow^* and homozygous for *Hbb^tm3(HBG1,HBB)Tow^*) were purchased from Jackson Laboratories (Bar Harbor, ME, USA). SCD mice were randomly divided into two groups of six: one group received only control chow and another group only GBT1118-containing chow. Non-SCD mice also received the control chow. GBT1118-containing chow and control chow were provided by Global Blood Therapeutics (Pfizer) (New York, NY, USA) with dosing as described previously [13,14]. Mice were acclimatized to the vivarium for one week. At the seven-week age, peripheral blood was collected on Day 0 as the baseline and at 2 months and 4 months post-GBT1118 treatment from all mice. All mice were subjected to 12 h water deprivation for seven consecutive days every three weeks to induce VOC [14,15]. Mice were euthanized after 4 months of GBT1118 treatment (Figure 1). After dissecting the whole liver from euthanized mice, it was rinsed in cold PBS, lightly blotted dry on lint-free paper, and then processed for all experiments. All animal experiments were approved by the Institutional Animal Care and Use Committee at the SUNY Upstate Medical University (Protocol # 510).

### 2.2. Blood and Serum Analysis

Complete blood count (CBC) was performed using Abaxis Vetscan HM5 v2.3 hematology analyzer (Union City, CA, USA). Alkaline phosphatase (ALP), alanine transaminase (ALT), total bilirubin (TBIL), and bile acid were measured in serum using a mammalian liver profile reagent rotor with the Zoetis Vetscan VS2 chemistry analyzer (Parsippany-Troy Hills, NJ, USA).

### 2.3. Protein Extraction

Total protein from liver was extracted using RIPA lysis buffer (AAJ62524AD; Thermo Fisher, Waltham, MA, USA) supplemented with protease inhibitors (PIA32965; Pierce, Waltham, MA, USA) and quantified using a BCA protein assay kit (PIA53226; Pierce, Waltham, MA, USA).

### 2.4. ELISA

Hemopexin was measured in serum (OKIA00097; Aviva Systems Biology, San Diego, CA, USA) and *malondialdehyde* (MDA) (STA-832; Cell Biolabs, San Diego, CA, USA) in liver protein lysate by enzyme-linked immunosorbent assays (ELISA) kits.

### 2.5. RNA Extraction, Reverse Transcription and qPCR

Liver total RNA was extracted using TRIzol (15596026; Life Technologies, Carlsbad, CA, USA). Total RNA (2.5 µg) was used to make complementary DNA (cDNA) with SuperScript IV^®^ First-Strand Synthesis kit (18091050; Life Technologies, Carlsbad, CA, USA) using random hexamers as the primer. For quantification of mRNA abundance, qPCR was performed using PowerTrack SYBR Green Master Mix (A46109; Thermo Fisher, Waltham, MA, USA) in Bio-Rad’s CFX Connect Real-Time PCR Detection System (Hercules, CA, USA). Gene-specific primers are listed in Table 1. Relative mRNA levels were normalized to 18S rRNA using the 2^−ΔΔC(T)^ method.

### 2.6. Histology and Immunohistochemistry

Liver dissected out of euthanized mice were rinsed in cold PBS to remove blood and then fixed in neutral buffered formalin (10%) at a 1:10 ratio of tissue volume to fixative volume for 48 h at 4 °C. Fixed paraffin-embedded 5 µ tissue sections were stained with hematoxylin and eosin (H&E), Perls *Prussian* blue stain kit (24199-1; Polysciences, Warrington, DC, USA) for iron, and *Masson’s trichrome* stain kit (9179A; Newcomer Supply, Middleton, WI, USA) for connective tissue indicting fibrosis. Terminal deoxynucleotidyl transferase dUTP nick-end labeling (TUNEL) assay (ab206386; Abcam, Waltham, MA, USA) was performed following kit instructions. 4-Hydroxynonenal (4-HNE) (Abcam, ab46545, 1:200) for ferroptosis was detected after citrate (pH 6) antigen retrieval. Horseradish peroxidase (HRP)-conjugated goat anti-rabbit secondary antibody (Invitrogen, 31460, 1:500) was detected with metal enhanced 3,3-diaminobenzidine (DAB) substrate kit (34065; Thermo Scientific, Waltham, MA, USA). Methyl green was used as a counterstain. Images were captured using an Aperio digital slide scanner (Leica Biosystems, Deer Park, TX, USA) and analyzed using Aperio ImageScope software v12.4.6 (Leica Biosystems, Deer Park, TX, USA). To quantify apoptosis, 4-HNE staining, Perls Prussian blue stain, fibrosis and necrosis area, 10 spots were randomly selected from each tissue section from each animal.

### 2.7. Statistical Analyses

Data were presented as mean ± standard deviation. For multiple comparisons, 2-way repeated measures analysis of variance (ANOVA) with a Bonferroni post hoc test was used. The statistical significance of two groups was evaluated using the two-sided Mann–Whitney test. Differences with *p* ≤ 0.05 were considered significant. All statistical analyses were conducted with GraphPad Prism software version 5.

## 3. Results

### 3.1. GBT1118 Reduces Erythrocyte Hemolysis, Improves Hemoglobin and Reduces Splenomegaly

**Figure 2:**
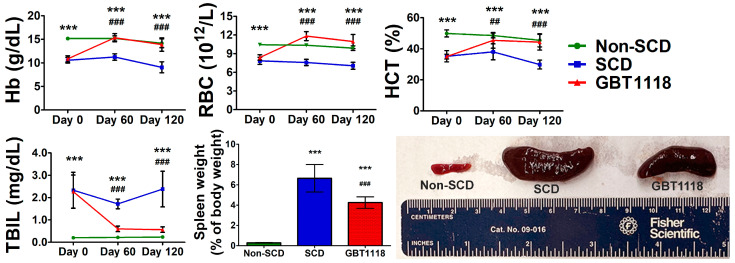
Hemoglobin (Hb) levels, red blood cell (RBC) counts, hematocrit (HCT) percentage, total bilirubin (TBIL) concentration and spleen weight and size at Day 120. N = 6, (***) *p* < 0.001 for non-SCD compared to SCD and GBT1118. (##) *p* < 0.01, (###) *p* < 0.001 for GBT1118 compared to SCD using 2-way repeated measures ANOVA with Bonferroni post hoc multiple comparison test.

GBT1118 prevents erythrocyte sickling by decreasing the overall deoxyHb concentration [12] to ameliorate the unwanted consequences resulting from sustained sickling. As reported previously by our group and others [11,12,13,14], GBT1118 treatment resulted in an increase in total Hb, red blood cell (RBC) counts and hematocrit (HCT) percentages in SCD mice, comparable to those in non-SCD mice (Figure 2; Supplementary Data). In addition, total bilirubin (TBIL) levels were also significantly improved in SCD mice after GBT1118 treatment. Bilirubin levels correlate with lactic dehydrogenase, which is related to the degree of hemolysis, ineffective erythropoiesis or both. These results are consistent with the observations in SCD patients treated with voxelotor [9]. Concurrent increases in Hb, RBC counts and HCT percentage and a reduction in TBIL demonstrate reduced erythrocyte hemolysis. Improvements in hemodynamics due to GBT1118 use were maintained throughout the treatment period. Sequestration of sickled erythrocytes, obstruction in splanchnic vasculature and chronic hemolysis leads to splenomegaly in SCD. With the reduction in sickling and hemolysis, SCD mice treated with GBT1118 showed reduced spleen weight and size (Figure 2).

### 3.2. GBT1118 Improves Liver Function

**Figure 3:**
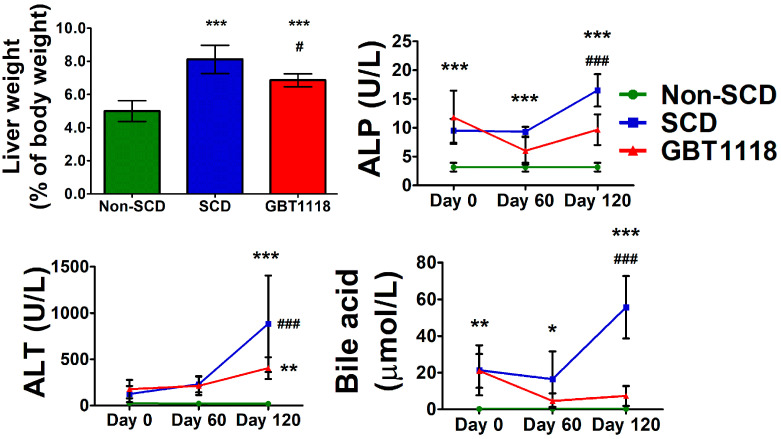
Liver weights in non-SCD, SCD and GBT1118-treated SCD mice after 4 months of treatment, and levels of alkaline phosphatase (ALP), alanine transaminase (ALT), and bile acid at baseline, 2 months and 4 months. N = 6, (*) *p* < 0.05, (**) *p* < 0.01, (***) *p* < 0.001 for non-SCD compared to SCD and GBT1118. (#) *p* < 0.05, (###) *p* < 0.001 for GBT1118 compared to SCD using 2-way repeated measures ANOVA with Bonferroni post hoc multiple comparison test.

Sequestration of sickled erythrocytes in the liver and splanchnic vasculature causes an increase in the size of the organ. Hepatomegaly was observed in 91 percent of 70 SCD patients who underwent autopsy [16]. Intrahepatic sickling and hypoxic hepatocellular damage lead to defects in liver function. While liver weights in SCD and GBT1118-treated SCD mice were significantly increased compared to untreated SCD mice, GBT1118 treatment was associated with reduced liver weight in SCD mice (Figure 3). It is known that voxelotor is metabolized in the liver by the CYP 3A4 and to some extent by CYP2B6, CYP2C9 and CYP2C19 [17]. Their intermediate metabolic product may cause liver injury and an increase in weight [18]. However, the exact mechanism here remains speculative. Liver function tests in SCD were much improved after GBT1118 treatment. Liver enzymes alkaline phosphatase (ALP), alanine transaminase (ALT), and bile acids, which worsened with age, were higher in SCD mice than non-SCD mice (Figure 3). The levels of ALP, ALT and bile acids are elevated in SCD and increase even further during VOC [19]. Increased ALT reflects hepatocyte injury, and elevated ALP and bile acids indicate cholestasis. While GBT1118-treated SCD mice exhibited lower ALP, ALT and bile acids, they still remained significantly higher compared to non-SCD mice.

### 3.3. Liver Inflammation in SCD Is Reduced with GBT1118 Treatment

**Figure 4:**
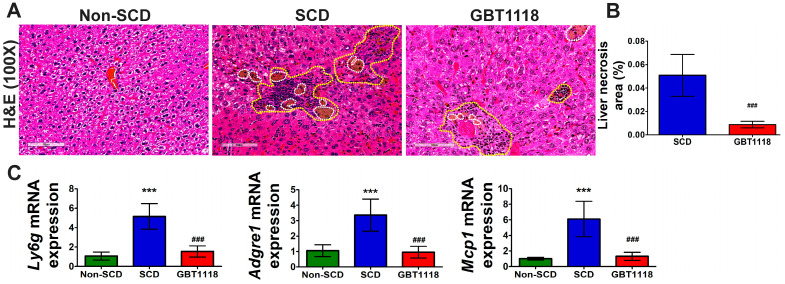
(**A**) Leukocyte infiltration, and (**B**) necrosis area in liver tissues, quantified by hematoxylin and eosin (H&E) staining. Area within yellow-colored dashed line shows leukocyte infiltration, and area within white-colored dashed line shows necrosis. Scale bar 100 μm; (**C**) inflammatory cell marker infiltrated in the liver quantified by qPCR using mRNAs for lymphocyte antigen 6 family member G (*Ly6g*), monocyte chemoattractant protein 1 (*Mcp1*), and adhesion G protein-coupled receptor E1 (*Adgre1)* gene encoding F4/80 protein in non-SCD, SCD and GBT1118-treated SCD mice at 4 months post-treatment. qPCR mRNA values normalized to 18S rRNA and compared using 2-way repeated measures ANOVA with Bonferroni post hoc multiple comparison test. Necrosis area percentage was evaluated using Mann–Whitney test. N = 6, (***) *p* < 0.001, for non-SCD compared to SCD and GBT1118. (###) *p* < 0.001 for GBT1118 compared to SCD.

SCD is associated with chronic inflammation that is exasperated during VOC. Higher leukocyte counts, elevated levels of circulating activated and aged neutrophils (CANs), monocytes and activated platelets are attributed to increased hemolysis-derived inflammation in SCD [20]. Systemic inflammation, along with chronic hemolysis, contributes to acute hepatic crisis, which triggers liver inflammation and parenchymal necrosis. High inflammation causes leukocyte infiltration into target organs [21,22]. There was a consistent increase in inflammatory cells, leukocyte infiltration and necrosis (Figure 4A,B) in SCD liver. The presence of inflammatory cells in untreated SCD liver was confirmed by the higher expression of mRNAs for multiple inflammatory cell markers such as lymphocyte antigen 6 family member G (*Ly6g*), monocyte chemoattractant protein-1 (*Mcp1*), and adhesion G protein-coupled receptor E1 (*Adgre1)* gene encoding F4/80 protein (Figure 4C). GBT1118 treatment resulted in reduced liver inflammation, leukocyte infiltration and necrosis in SCD mice.

### 3.4. Liver Heme and Iron Homeostasis in SCD Are Improved after GBT1118 Treatment

**Figure 5:**
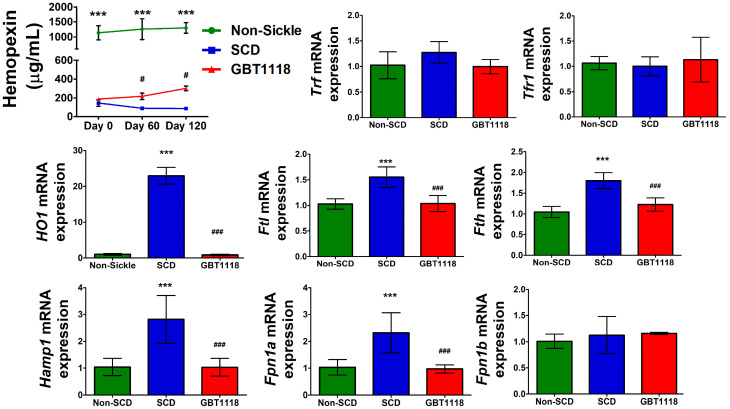
Serum hemopexin concentration, and expression of mRNAs for genes regulating iron homeostasis in the liver, transferrin (*Trf*), transferrin receptor 1 (*Tfr1*), heme oxygenase 1 (*Hmox1*), ferritin L (*Ftl*) and ferritin H (*Fth*) hepcidin (*Hamp1*), ferroportin 1a (*Fpn1a*) and ferroportin 1b (*Fpn1b*). qPCR mRNA values normalized to 18S rRNA using 2-way repeated measures ANOVA with Bonferroni post hoc multiple comparison test. N = 6, (***) *p* < 0.001 for non-SCD compared to SCD and GBT1118. (#) *p* < 0.05, (###) *p* < 0.001 for GBT1118 compared to SCD.

The presence of excess heme in SCD occurs due to increased hemolysis [23]. This reduces the heme scavenger protein, hemopexin (Figure 5). With the reduction in hemolysis, as reflected by the rise in Hb and lower TBIL, there was a significant increase in serum hemopexin after GBT1118 treatment. However, this increase in hemopexin in GBT1118-treated mice was not comparable to non-SCD mice and remained significantly lower.

Excess heme and reduction in hemopexin may perturb iron homeostasis in SCD. Proteins synthesized in the liver help in maintaining iron homeostasis. While hepcidin and ferroportin regulate iron metabolism, transferrin and transferrin receptors control iron circulation and cellular uptake [24]. There was no difference in transferrin (*Trf*) and transferrin receptor 1 (*Tfr1*) mRNA expression between untreated SCD mice and GBT1118-treated mice, and both were comparable to non-SCD mice (Figure 5). Expression of mRNAs of other iron regulatory proteins heme oxygenase-1 (*Hmox1*), ferritin-L (*Ftl*) and ferritin-H (*Fth*), hepcidin (*Hamp1*) and ferroportin isoform 1a (*Fpn1a*) were significantly higher in SCD liver. Another mRNA isoform of ferroportin, *Fpn1b*, did not change in any group. Except *Trf* and *Tfr1*, GBT1118 treatment reversed mRNA expression of all other iron regulatory proteins in SCD mice livers.

### 3.5. Apoptosis and Fibrosis in SCD Are Reduced after GBT1118 Treatment

**Figure 6:**
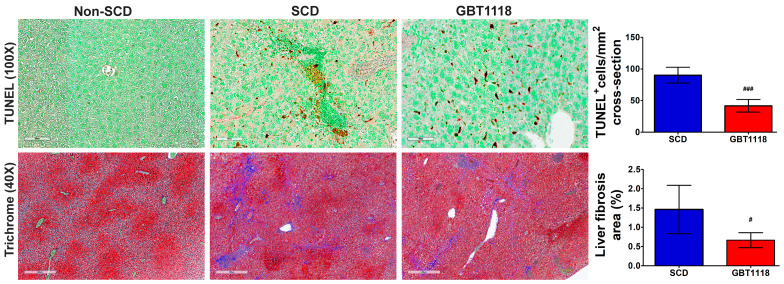
Apoptosis quantified by *TUNEL* staining in liver tissues. Apoptotic nuclei (brown stain) detected using 3,3-diaminobenzidine (DAB). Tissues counterstained with methyl green. Scale bar, 100 μm; fibrosis quantified by Masson’s trichrome staining in liver tissues. Scale bar, 400 μm. N = 6, for non-SCD compared to SCD and GBT1118. (#) *p* < 0.05, (###) *p* < 0.001 for GBT1118 compared to SCD using Mann–Whitney test.

Liver damage in SCD may occur due to apoptosis. There was a significant increase in TUNEL-positive nuclei in untreated SCD liver compared to non-SCD mice liver (Figure 6). However, GBT1118 treatment resulted in a significant decrease in apoptosis in the liver of SCD mice. Recurrent I/R injury with parenchymal necrosis, high inflammation and apoptosis can also induce fibrosis, which is common in SCD patients with hepatopathy [25]. Moreover, hepatic cirrhosis patients have increased levels of iron and ferroptosis markers of lipid peroxidation [26]. Consistent with the development of liver cirrhosis, there was an increase in fibrosis in untreated SCD mice liver, which was reduced significantly in GBT1118-treated mice (Figure 6).

### 3.6. GBT1118 Treatment Resulted in Reduced Iron Overload and Decreased Liver Ferroptosis

**Figure 7:**
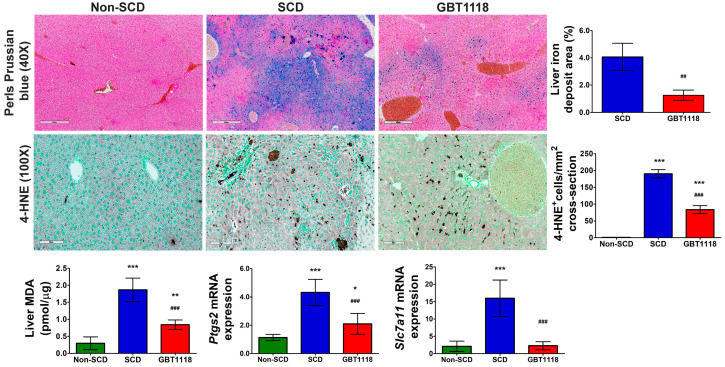
Perls Prussian blue stain in liver sections. Scale bar, 400 µm, and quantification of iron deposit area percentage; 4-hydroxynonenal (4-HNE) in the liver. Scale bar: 100 μm. Quantification of 4-HNE stained cells, *Malondialdehyde* (MDA) concentration, and expression of ferroptotic markers *Ptgs2* and *Slc7a11* mRNAs in the liver. qPCR mRNA values normalized to 18S rRNA and compared using 2-way repeated measures ANOVA with Bonferroni post hoc multiple comparison test. Iron deposit area percentage was evaluated using Mann–Whitney test N = 6, (*) *p* < 0.05 (**) *p* < 0.01, (***) *p* < 0.001 for non-SCD compared to SCD and GBT1118. (##) *p* < 0.01, (###) *p* < 0.001 for GBT1118 compared to SCD.

Perturbation in iron homeostasis was reflected by an increase in iron overload detected by Perls Prussian blue staining in the liver of SCD mice (Figure 7). The iron overload may trigger ferroptosis. There were increases in several putative ferroptosis markers, including lipid peroxidation-derived 4-hydroxynonenal (4-HNE) staining (Figure 7) [27], malondialdehyde (MDA) levels [28], and prostaglandin-endoperoxide synthase 2 (*Ptgs2*) [28] and solute carrier family 7, member 11 (*Slc7a11*) [28] mRNA levels in SCD mice liver (Figure 7). Adjacent sections stained by H&E and for 4-HNE revealed an overlap of cells having necrotic morphotype (Figure 7) indicating ferroptotic cell death of hepatocytes and immune cells, possibly macrophages (Figure 7). GBT1118-treated mice exhibited reduced ferroptosis markers. *Slc7a11* mRNA expression returned to normal levels, and MDA concentration, 4-HNE staining and *Ptgs2* mRNA expression were significantly lower than untreated SCD mice, although still higher than non-SCD mice.

## 4. Discussion

In this study, Townes SCD mouse models—that recapitulate SCD in humans—were treated with GBT1118 to investigate the effects of the voxelotor on hepatopathy associated with the disease. We first demonstrated that there were higher inflammation and alterations in liver function in these mice. We also showed that the disruption in iron homeostasis in the liver of SCD mice was attenuated by GBT1118. This was associated with lower iron overload and ferroptosis in the liver. In addition, we found less apoptosis and fibrosis in the liver of SDC mice after 4 months of GBT1118 treatment, compared with untreated SCD mice. Since GBT1118 is a Hb modifier that inhibits erythrocyte sickling, the improvements in liver pathophysiology observed in the GBT1118-treated SCD mice were related to less free heme due to lower Hb sickling and less hemolysis [13], as reflected by the increase in Hb, RBC counts and HCT percentage.

Hemolysis and free heme can cause inflammation and liver injury. The iron derived from excessive heme triggers inflammation by neutrophil activation [29] and the release of neutrophil extracellular traps (NETs) from CANs [30]. The iron moiety in heme, via Toll-like receptor 4 (TLR4) signaling and reactive oxygen species (ROS), alters the macrophage phenotype toward the M1-like proinflammatory phenotype [31,32]. Proinflammatory macrophages, along with the nucleotide-binding domain and leucine-rich repeat-containing family, pyrin domain containing 3 (NLRP3) inflammasome components, and IL-1R, contribute to SCD inflammation [33]. These inflammatory processes are a major contributing factor of VOC in SCD [34]. Following the resolution of VOC-induced sinusoidal ischemia, reperfusion exacerbates liver injury. Hepatocyte injury triggers inflammatory reaction [35]. In addition, improvement in intestinal pathophysiology may also reduce liver inflammation in GBT1118-treated mice. Chronic intestinal hypoxia in SCD, especially after VOC, causes dysbiosis and an increase in microbial load [15,36]. These changes were associated with the disruption of intestinal tight junctions and increased circulating pathogen-associated molecular pattern molecules (PAMPs), such as lipopolysaccharide (LPS) [36], that regulate CANs [34]. GBT1118 improves blood flow in the splanchnic vasculature by reducing sickling and hemolysis of erythrocytes. This not only helps maintain proper oxygenation of enterocytes but also decreases intestinal leakiness and the release of PAMPs that trigger CANs [14]. All these mechanisms collectively contribute to the reduction in inflammation.

The regulation of the expression of various iron metabolism genes in the liver helps in maintaining iron homeostasis. Due to persistent hemolysis, free Hb and excess heme, along with the depletion of hemopexin, the heme scavenger causes tissue oxidative injury and triggers inflammation in SCD [37]. Hemopexin is produced in the liver and has a very high affinity for heme (Kd < 10^−13^ M) making the complex virtually irreversible [38]. Heme bound to hemopexin is transported for catabolism to prevent heme-mediated oxidative damage. The heme–hemopexin complex binds to the LDL receptor-related protein-1 (LRP1/CD91) for endocytosis [39]. While some hemopexin is recycled, the majority is degraded. This creates an acquired hemopexin deficiency, especially during massive hemolysis [40], as observed in SCD. Under hemolytic stress, hemopexin deficiency led to acute kidney injury in SCD mice, which was protected by pretreatment with purified hemopexin [41]. Hemopexin therapy also improves cardiovascular function by preventing heme-induced endothelial toxicity in SCD [42]. Chronic hemolysis in SCD increases heme oxygenase-1 (HO-1) [43]. The transport of the heme–hemopexin complex into the liver triggers the expression of HO-1 and ferritin. HO-1 catabolizes heme to generate biliverdin, ferrous iron (Fe^2+^) and carbon monoxide [44]. Enhanced HO-1 activity contributes to the increase in cellular iron levels and promotes ferritin synthesis to sequester iron [45]. Stress and inflammation also upregulate *Hmox1*. While moderate levels of HO-1 activation are considered cytoprotective in various pathological conditions [46], overactivation of HO-1 can be cytotoxic due to excessive increase in labile Fe^2+^ beyond the buffering capacity of ferritin [45].

Additional stress in the liver due to the increase in the availability of excess iron may alter iron metabolism. Hepcidin expression increases when the body has excess iron resulting in iron storage in hepatocytes [47]. Moreover, inflammation can trigger hepcidin expression. Inflammation causes sequestration of iron in tissues, and pro-inflammatory cytokines, such as interleukin-6 (IL-6), enhance *Hamp1* transcription [48]. Iron homeostasis is regulated by hepcidin that binds to ferroportin to internalize and degrade ferroportin, resulting in cellular iron retention and decreased iron exports. Here, while the expression of *Fpn1a* was elevated in SCD and decreased with GBT1118 treatment, the isoform *Fpn1b* did not change. The regulation of ferroportin expression is complex. In the duodenum and during erythropoiesis, iron deficiency increases the expression of the ferroportin splice variant *Fpn1b* [49]. However, in the liver, ferroportin expression goes up due to high levels of iron [50]. The isoform *Fpn1a* contains an iron response element (IRE) at its 5’ region. In erythrophagocytosing macrophages, iron liberated from heme by HO-1 inactivates iron regulatory proteins (IRP), resulting in *Fpn1a* expression [49]. In addition, under low cellular ion levels, miR-485-3p microRNA targets the 3’ untranslated region of ferroportin to downregulate it [51]. With an increase in cellular iron, this miRNA expression is reduced, which helps increase ferroportin expression. On the contrary, *Fpn1b*, which produces an identical protein, lacks the 5’ IRE [49]. Consequently, the IRE/IRP system is unable to exert its influence on the expression of ferroportin.

Excess free heme and increased free iron, a product of heme degradation through HO-1, and perturbed iron homeostasis may contribute to liver iron overload in SCD mice. Iron overload was significantly reduced in GBT1118-treated mice liver. While excessive iron storage in human SCD patients is often correlated with multiple blood transfusions and a rise in serum ferritin, SCD mice liver had iron overload independent of any blood transfusion. It is known that frequent VOC causes liver sinusoidal ischemia resulting in iron accumulation and hepatic injury [7]. Intracellular iron accumulation also triggers inflammation [1], which creates a vicious cycle of iron-induced oxidative stress.

The production of ROS can directly oxidize various biomolecules and inactivate enzymes. This triggers a cascade of events leading to apoptosis, increased proinflammatory signaling, modification of the expression of adhesion molecules on the surface of leukocytes and endothelial cells, and promotion of nitric oxide biodeficiency [52,53]. Tissue hypoxia and ROS production during I/R activate and recruit cells of the innate and adaptive immune system and release proinflammatory mediators in the site of ischemic to induce apoptosis and fibrosis. In SCD liver, the lack of hypoxia-induced nuclear factor-κB p65 activation, along with the imbalance in the endothelial/inducible nitric oxide synthase response to I/R injury and the inability to increase hypoxia-induced expression of HO-1/biliverdin reductase, contribute to liver injury that leads to apoptosis and fibrosis [54]. As cells die, apoptotic cells are cleared by efferocytosis where macrophages are recruited for the clearance. Excess heme drives the activation of TLR4 signaling and reduces the expression of metabolic transcription factors, such as peroxisome proliferator-activated receptor γ coactivator 1α (PGC1α) and proliferator-activated receptor γ (PPARγ), to inhibit efferocytosis [55]. This enables macrophages to recruit immune cells, switch resident macrophages from an anti-inflammatory to a pro-inflammatory state, and recruit monocytes/neutrophils to the damaged site of the liver. These events, as a result of excess heme, promote apoptosis and fibrosis in the liver.

Iron overload may also cause liver injury by ferroptosis [26]. Ferroptosis is a regulated cell death process that is characterized by iron-dependent lipid peroxidation [56]. MDA and 4-HNE are toxic derivatives of decomposed lipid peroxides produced due to lipid peroxidation triggered by an imbalance in iron homeostasis. These byproducts of lipid peroxides react with DNA bases, proteins, and other nucleophilic molecules, amplifying ROS signaling and resulting in ferroptosis [56]. Overexpression of *Ptgs2* is considered a biomarker of ferroptosis and is related to increased ferroptosis [57]. SLC7A11 is a member of the subunit of system χ_c_^−^, which is a cystine/glutamate antiporter mediating the efflux of cellular glutamate and the influx of cystine [58]. Inside the cell, cystine is reduced to cysteine during the synthesis of glutathione (GSH) [59]. GSH is an ROS scavenger, and reducing GSH levels by deleting the enzyme glutamate cysteine ligase induces ferroptosis [60]. In a mouse model of hereditary hemochromatosis, iron overload-induced ferroptosis in the liver was associated with the increase in *Slc7a11* expression [28]. *Slc7a11* upregulation is mediated by iron through the ROS-nuclear factor erythroid 2-related factor 2 (NRF2)-antioxidant response element (ARE) axis. *Hmox1* and ferroportin expressions are also under the regulation of *Nrf2* [44,49]. Both genes contain the ARE in their promoter sequences. AREs can be bound either by the repressor BTB and CNC homology 1 (BACH1) or the transcription activator NRF2 [49]. Heme degrades BACH1 and allows NRF2 to activate the transcription of *Hmox1* and ferroportin. *Hmox1* can also be transcriptionally upregulated by 4-HNE [61] to induce ferroptosis, as observed in cardiomyopathy in SCD [23]. Thus, by reducing hemolysis and free heme, ferroptosis may have been reduced in SCD mice liver after GBT1118 treatment.

In addition to ferroptosis, increased apoptotic cell death in SCD mice liver was ameliorated by GBT1118 treatment. This is likely the result of hepatic I/R injury and inflammation [25]. Ferroptosis may also cause increased cell apoptosis [62]. Tumor protein 53 (*p53*) plays a pivotal role in the interplay between ferroptosis and apoptosis. Stress-activated *p53* induces apoptosis [63]. It can also regulate the expression of *Ptgs2* and promote ferroptosis [64]. The inhibitor of the apoptosis-stimulating protein of p53 (iASPP) can repress p53-induced apoptosis as well as inhibit ferroptosis by regulating the NRF2 pathway [65]. Reduction in inflammation, iron overload, ferroptosis and apoptosis may have led to the lowering of liver fibrosis in SCD mice after GBT1118 treatment.

## 5. Conclusions

Our study shows that by lowering hemolysis and reducing the availability of free heme, GBT1118 reverses the imbalance in tissue iron metabolism by increasing hemopexin and reducing HO-1. As a result, there was less iron overload and inflammation and decreased hepcidin, ferritin and ferroportin expression, which protects SCD liver from injury by reducing apoptosis, fibrosis and ferroptosis. These findings provide the molecular basis for liver ferroptosis, which contributes to sickle hepatopathy, and demonstrate that GBT1118 can ameliorate chronic liver disease in SCD mice. Recurrent blood transfusions in SCD patients increase the risk of iron overload in the liver. Chronically transfused SCD patients with iron overload have a significantly higher rate of mortality compared to those without iron overload [66]. It would be interesting to examine whether the voxelotor has similar beneficial effects on the liver of SCD patients, especially those who have iron overload due to blood transfusion.

## Figures and Tables

**Table 1:** List of qPCR primers.

Gene	Forward	Reverse
*Slc7a11*	TTCATCCCGGCACTATTTTC	CGTCTGAACCACTTGGGTTT
*Ptgs2*	CTGCGCCTTTTCAAGGATGG	GGGGATACACCTCTCCACCA
*Hmox1*	GCCGAGAATGCTGAGTTCAT	TCCAGGGCCGTGTAGATATG
*Trf*	TTCTGTAAGCTGTCGGAGCC	GACACAACTGCCCGAGAAGA
*Tfr1*	TGGCTGAAACGGAGGAGACAGA	TGGCTCAGCTGCTTGATGGTGT
*Ftl*	CGGGCCTCCTACACCTACCT	CCCTCCAGAGCCACGTCAT
*Fth*	AAGATGGGTGCCCCTGAAG	CCAGGGTGTGCTTGTCAAAGA
*Hamp1*	GCACCACCTATCTCCATCAACA	TTCTTCCCCGTGCAAAGG
*Ly6g*	TTGCAAAGTCCTGTGTGCTC	AGGGGCAGGTAGTTGTGTTG
*Mcp1*	AGGTCCCTGTCATGCTTCTG	TCTGGACCCATTCCTTCTTG
*Adgre1*	CTTTGGCTATGGGCTTCCAGTC	GCAAGGAGGACAGAGTTTATCGTG
*Fpn1a*	AAAGAAGACCCCGTGACAGC	TCCCCGTGTTTGTTCTGATG
*Fpn1b*	GCCGGTTGGAGTTTCAATGT	TCCCCGTGTTTGTTCTGATG
*18S rRNA*	GTAACCCGTTGAACCCCATT	CCATCCAATCGGTAGTAGCG

## Data Availability

Data are available from the corresponding author upon reasonable request.

## Supplementary Materials

The following supporting information can be downloaded at: https://www.mdpi.com/article/10.3390/medicina60101581/s1.
