# Supplementary material for: GBT1118, a Voxelotor Analog, Ameliorates Hepatopathy in Sickle Cell Disease
Source: Medicina (Kaunas). 2024 Sep 26;60(10):1581. doi: 10.3390/medicina60101581 (PMC11509622; doi:10.3390/medicina60101581)
Supplement: Supplementary file 1 [file medicina-60-01581-s001.zip › medicina-3202029-supplementary.pdf]

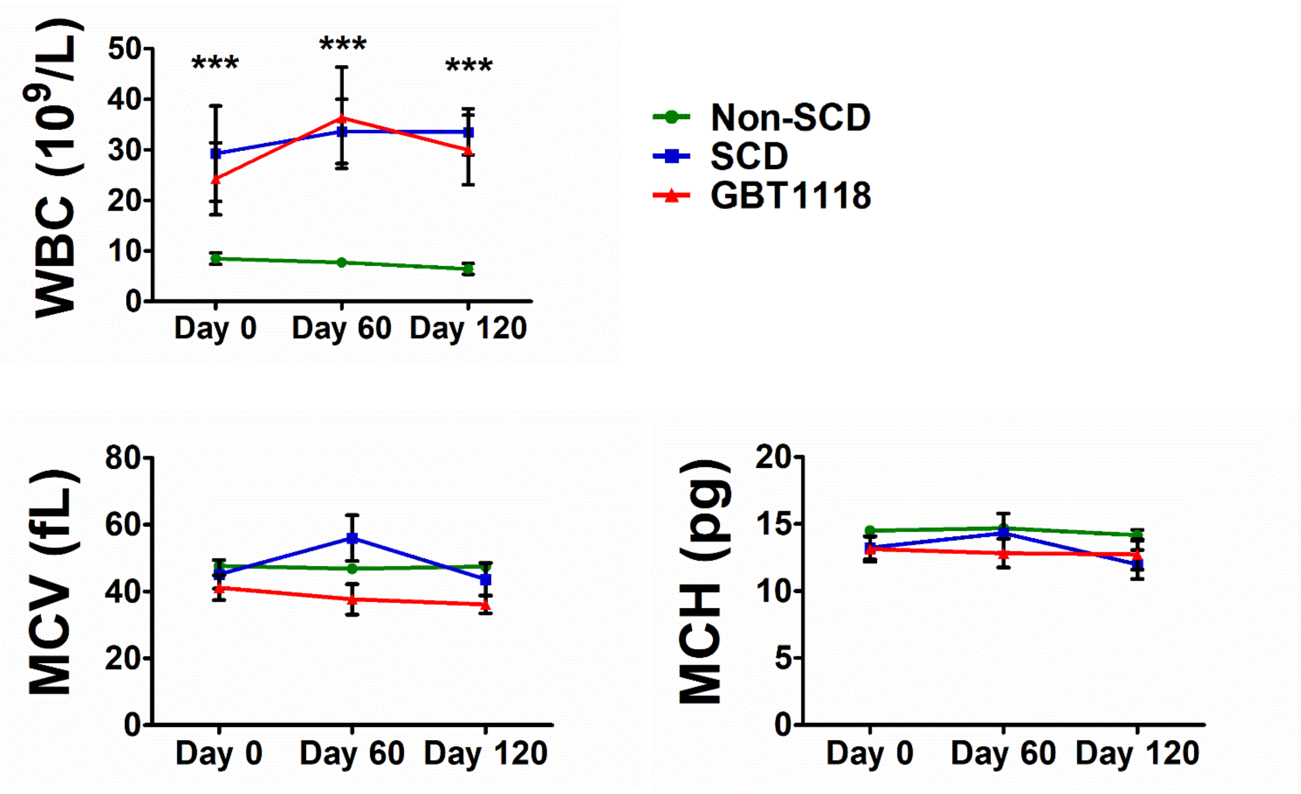

Supplement Figure S1: White blood cell (WBC) count; Mean corpuscular volume (MCV); and Mean corpuscular hemoglobin (MCH) in peripheral blood of non-SCD, SCD and GBT1118 groups, N=6. (***)p<0.001 for non-SCD compared to SCD and GBT1118.
